# Analysis of thromboelastography, PT, APTT and fibrinogen in intraosseous and venous samples—an experimental study

**DOI:** 10.1186/s13049-016-0318-0

**Published:** 2016-11-03

**Authors:** Gunnar Strandberg, Miklós Lipcsey, Mats Eriksson, Norbert Lubenow, Anders Larsson

**Affiliations:** 1 Anaesthesiology and Intensive Care, Department of Surgical Sciences, Uppsala University, Uppsala, Sweden; 2 Hedenstierna laboratory, Anaesthesiology and Intensive Care, Department of Surgical Sciences, Uppsala University, Uppsala, Sweden; 3 Immunology and Transfusion medicine, Department of Immunology, Genetics and Pathology, Uppsala University, Uppsala, Sweden; 4 Clinical Chemistry, Department of Medical Sciences, Uppsala University, Uppsala, Sweden; 5 Department of Surgical Sciences, Uppsala University, Uppsala University Hospital, 751 85 Uppsala, Sweden

**Keywords:** Blood coagulation, Haemorrhage, Infusions, Intraosseous, Thrombelastography

## Abstract

**Background:**

Laboratory analysis of coagulation is often important in emergencies. If vascular access is challenging, intraosseous catheterization may be necessary for treatment. We studied the analysis of coagulation parameters in intraosseous aspirate during stable conditions and after major haemorrhage in a porcine model.

**Methods:**

Ten anesthetized pigs received central venous and intraosseous catheters and samples were taken for analysis of thromboelastography (TEG), prothrombin time (PT), activated partial thromboplastin time (APTT) and fibrinogen concentration. Analyses were repeated after removal of 50 % of the calculated blood volume and resuscitation with crystalloid. Intraosseous and venous values were compared.

**Results:**

Bleeding and resuscitation resulted in haemodilution and hypotension. Median TEG reaction time was shorter in intraosseous than in venous samples before (1.6 vs 4.6 min) and after (1.6 vs 4.7 min) haemodilution. Median maximal amplitude was smaller in intraosseous samples at baseline (68.3 vs 76.4 mm). No major differences were demonstrated for the other TEG parameters. The intraosseous samples often coagulated in vitro, making analysis of PT, APTT and fibrinogen difficult. After haemodilution, TEG maximal amplitude and α-angle, and fibrinogen concentration, were decreased and PT increased.

**Discussion:**

The intraosseous samples were clinically hypercoagulable and the TEG demonstrated a shortened reaction time. The reason for this may hypothetically be found in the composition of the IO aspirate or in the sampling technique. After 50 % haemorrhage and haemodilution, a clinically relevant decrease in fibrinogen concentration and a lower TEG maximal amplitude were observed.

**Conclusions:**

Although the sample is small, these data indicate that intraosseous samples are hypercoagulable, which may limit their usefulness for coagulation studies. Major haemodilution only moderately affected the studied parameters.

**Electronic supplementary material:**

The online version of this article (doi:10.1186/s13049-016-0318-0) contains supplementary material, which is available to authorized users.

## Background

Laboratory analysis of coagulation provides important information in many emergencies where coagulopathies are common, including trauma, major surgery and sepsis, and in the large population of patients using medications affecting this system [1–3].

In the critically ill patient where intravenous access is unsuccessful, intraosseous access is recommended [4, 5]. Intraosseous access has been demonstrated to be a fast, reliable and safe method that permits administration of drugs and resuscitation fluids in various settings [6, 7]. In an emergency, questions may arise whether blood from an intraosseous catheter can also be used for laboratory analyses, and this has been the subject of some investigation [8, 9].

For monitoring of coagulation, the platelet count, prothrombin time (PT), activated partial thromboplastin time (APTT) and plasma fibrinogen concentration are frequently studied parameters. A rising interest is also seen for global point of care methods, i.e. thromboelastography (TEG) / thromboelastometry, as monitors of coagulation in emergencies and in surgical haemorrhage [2, 10, 11].

Previous data suggest that platelet counts in intraosseous aspirate do not adequately reflect levels in venous blood and there may also be a potential risk that bone marrow debris in the samples could damage cell counters [12–14]. In addition to a different platelet content, bone marrow also contains lipid, and these factors could be hypothesized to affect coagulation analyses [15, 16].

The aim of this study was to evaluate whether aspirate from an intraosseous catheter could be used for TEG and standard coagulation assays, and whether values were comparable to those in simultaneously aspirated venous blood under stable conditions and after major haemorrhage and resuscitation.

As it would be both ethically and practically difficult to compare intraosseous and venous laboratory samples in critically ill or injured patients in a standardized manner, an animal model was considered the best option for this study. Since the circulatory system of the pig is most similar to that of humans among the non-primates, a porcine model was chosen. Such models have also previously been considered suitable for coagulation research [17, 18].

## Methods

### Animals

Ten healthy domestic-breed pigs weighing between 23. 1 and 28. 5 kg (mean 26. 4 ± 1. 7 kg) were included in the study. All animals were handled according to the guidelines of the Swedish National Board for Laboratory Animals and the European Convention on Animal Care. The Animal Ethics Committee of Uppsala University, Sweden, approved the experiment (C155/14, date of approval 2014-10-24).

### Anaesthesia and preparatory procedures

Anaesthesia was induced by an injection of 6 mg × kg^−1^ tilétamin-zolazepam mixed with 2.2 mg × kg^−1^ xylazin in the neck muscles. General anaesthesia was maintained with a continuous infusion of 8 mg × kg^−1^ × h^−1^ of pentobarbital mixed with 1.6 mg × kg^−1^ × h^−1^ of rocuronium and 0.48 mg × kg^−1^ × h^−1^ of morphine.

After receiving a bolus dose of 20 mg of morphine and 100 mg of ketamine, animals were tracheotomised and mechanically ventilated using a Servo I ^®^ ventilator (Maquet Critical Care, Solna, Sweden). During the experiment, the animals received 8 mL × kg^−1^ × h^−1^ of a balanced solution containing 25 mg × mL^−1^ of glucose, and 7 mL × kg^−1^ × h^−1^ of Ringer Acetate. An arterial catheter was placed into a right cervical artery and a central venous catheter was introduced through the right external jugular vein into the superior caval vein. A Swan-Ganz catheter was introduced through the right external jugular vein into the pulmonary artery. A 15G IO cannula (EZ-IO^®^, Teleflex Medical, Morrisville, NC, USA) was inserted into the proximal tibia. Correct placement was verified by the needle standing without support, aspiration of blood and by an incision to the bone after finishing the experiment. A 13.5 Fr. dialysis catheter was inserted through the left external jugular vein. A minor vesicotomy was performed and a urinary catheter was inserted. After preparation procedures, a stabilization period of 30 min in supine position preceded the experiment.

### Protocol

At baseline, simultaneous samples were collected from the intraosseous (IO) and central venous (CV) catheters. Blood was collected using 5 cc plastic syringes and immediately transferred to sodium citrate tubes for TEG and citrate- theophylline- adenosine- dipyridamole (CTAD) tubes for analysis of PT, APTT and fibrinogen concentration. 2 ml of blood was wasted before taking the samples for analysis and the catheters were flushed with 2 ml of saline after sampling. Samples were manually transported to the laboratory.

After the baseline samples, 50 % of the total calculated blood volume of 67 ml/kg was aspirated through the left jugular venous catheter and replaced with an equal volume of Ringer Acetate [19]. In conjunction with this, a small dose of heparin was administrated intravenously in six of the animals to achieve pathologic APTT results and to study the effect of different doses on the TEG. After a period of equilibration, new IO and CV samples were collected and the TEG, PT, APTT and fibrinogen analyses were repeated. The IO catheters were not used for infusion between the samples. After finishing the experiment, the animals were euthanized by potassium chloride injection.

### Laboratory analysis

The TEG analysis was performed in recalcified, kaolin activated whole blood according to manufacturer’s instructions (TEG 5000^®^, Haemoscope, Niles, IL). Analysis was performed both with and without heparinase. TEG parameters R (reaction time), K (kinetics), α-angle, maximal amplitude (MA) and lysis at 30 min (Ly30) were analysed.

Activated partial thromboplastin time (APTT, reagent STA PTT automate 5), fibrinogen (clot method, reagent 00673) and prothrombin time (PT, reagent STA SPA +) were analysed with a STA-R^®^ instrument (Diagnostica Stago S.A.S, Asnières sur Seine, France) with reagents from the same manufacturer. The prothrombin time was reported as International Normalized Ratio (INR) values. The total coefficients of variation for the methods were 1.9 % at 34.6 s for APTT, 3.0 % at 3 g/L for fibrinogen and 1.1 % at INR 1.0 for the prothrombin time.

### Statistical analysis

Data were examined for normality. Median and interquartile range (IQR) / range for IO and CV values from each sampling time were calculated for the measured parameters. Average differences between simultaneous IO and CV samples from the same individuals were calculated and reported as median and IQR / range. Differences between simultaneous IO and CV samples, and between baseline and second samples, were also assessed with Wilcoxon signed-rank test. Excel 2013^®^ (Microsoft Corp. Redmond WA) and Statistica 13^®^ (Statsoft Inc. Tulsa, OK) were used for the calculations.

## Results

All animals were successfully catheterized and aspiration of adequate sample volumes from the intraosseous catheters was generally feasible although it required somewhat more time and negative pressure than aspiration from the central venous catheters. On a few occasions it was difficult to draw the second intraosseous sample. A new catheter was then placed on the contralateral side and used for the second sample. Also, on a few occasions, clotting was observed in the tubes for the IO samples which then were re-drawn immediately. Samples were delivered to the laboratories within 5 min from drawing. Four animals received no heparin, one received 500, three received 1000, one 2000 and one 2500 IU. Due to logistic problems, baseline samples for PT, APTT and fibrinogen are missing from 1 animal and second TEG values from 2 animals.

Significant haemodilution occurred between first and second sampling with a reduction in mean (SD) haemoglobin level from 100 (6) to 57 (7) g × L^−1^. Mean arterial pressure (MAP) was also reduced from (mean (SD)) 73 (15) to 52 (7) mm Hg.

TEG analyses were carried out on all delivered samples with and without heparinase, the heparinase analysis was used for comparison between IO and CV samples. Two of the IO TEG-curves, one at baseline and one at second sample, were markedly different from the others. In the first one, K could not be calculated as MA never reached 20 mm. This curve was excluded from analysis for presumed technical fault.

Average IO and CV values and differences between simultaneous IO and CV samples are presented in Tables 1 and 2.

**Table 1:** TEG reaction time (R), kinetics (K), α-angle (α), Maximal Amplitude (MA), Lysis at 30 min (LY30) in intraosseous (IO) and central venous (CV) samples at baseline and average differences between simultaneous samples IO–CV from the same individuals

Variable	Unit	*n* IO	*n* CV	IO	CV	IO–CV
R	min	9	10	1.6 (1.2–2-2)	4.6 (4.4–6.7)	−2.8 (−5.8–(−1.8))
K	min	9	10	0.9 (0.8–1.0)	1.0 (0.8–1.1)	0 (−0.1–0)
α	°	9	10	77.3 (75.6–78.8)	77.2 (74.3–78.5)	−0.4 (−1.5–0.1)
MA	mm	9	10	68.3 (68.2–71.3)	76.4 (71.9–77.8)	−8.9 (−9.6–(−2.6))
LY 30	%	9	10	3.2 (2.2–4.9)	3.2 (2.3–4.9)	0.7 (−1.5–0.9)

Values are median and interquartile range (Q1–Q3)

**Table 2:** TEG reaction time (R), kinetics (K), α-angle (α), Maximal Amplitude (MA), Lysis at 30 min (LY30) in intraosseous (IO) and central venous (CV) samples after haemodilution and average differences between simultaneous samples IO–CV from the same individuals

Variable	Unit	*n* IO	*n* CV	IO	CV	IO–CV
R	min	8	8	1.6 (1.3–2.2)	4.7 (4.4–5.3)	−2.8 (−3.5–(−2.3)
K	min	8	8	1.2 (0.9–1.7)	1.2 (1.1–1.4)	−0.1 (−0.3–0.7)
α	°	8	8	75.3 (67.5–77.9)	73 (71.6–74.9)	2.5 (−7.3–4.7)
MA	mm	8	8	64.6 (54.8–69.3)	67.3 (65.8–69.3)	−0.9 (−14.9–2.5)
LY 30	%	8	8	3 (1.2–5.4)	2.4 (1.2–6.4)	0.1 (−1.4–0.4)

Values are median and interquartile range (Q1–Q3)

The reaction time (R) was invariably shorter in intraosseous compared with venous samples at both baseline and second sampling. The clot formation parameters, K and α-angle, were similar in IO and CV samples, while the MA was smaller in the IO samples at the first sampling.

Heparin had a dose dependent effect on the TEG when performed without heparinase. In the animal that received 2500 IU, no TEG curve could be produced in either IO or CV samples. In the animal receiving 2000 IU, no TEG curve was produced in the CV sample while there was a 2 min R prolongation compared with the heparinase analysis in the IO sample. After 1000 IU, R was prolonged with, on average, 2.3 min in the CV and 0.1 min in the IO samples and in the animal receiving 500 IU it was prolonged with 1.2 min in the CV but unaffected in the IO sample.

Although no visible clots were observed in the tubes when delivered to the laboratories, in a majority of the IO samples, the PT, APTT and fibrinogen analyses could not be performed because of clotting observed during the handling of the samples in the laboratory. All CV samples were analysed successfully. Average IO and CV values, differences and the number of samples included in the analysis are presented in Tables 3 and 4.

**Table 3:** Prothrombin Time, International Normalized Ratio (PT, INR), Activated Partial Thromboplastin Time (APTT), and fibrinogen in intraosseous (IO) and central venous (CV) samples at baseline and average differences between simultaneous samples IO–CV from the same individuals

Variable	Unit	*n* IO	*n* CV	IO	CV	IO–CV
PT	INR	4	9	1 (0.9–1.1)	1.1 (1–1.2)	−0.1 (−0.1–0)
APTT	s	3	9	63 (21–200)	67 (24–100)	−3 (−18–133)
Fibrinogen	g / L	4	9	1.8 (0.8–3.3)	1.8 (1.2–3.3)	0 (−1–0.1)

Values are median and range

**Table 4:** Prothrombin Time, International Normalized Ratio (PT, INR), Activated Partial Thromboplastin Time (APTT), and fibrinogen in intraosseous (IO) and central venous (CV) samples after haemodilution and average differences between simultaneous samples IO–CV from the same individuals

Variable	Unit	*n* IO	*n* CV	IO	CV	IO–CV
PT	INR	3	10	1.2 (1.1–1.3)	1.2 (1–1.3)	−0.1 (−0.1–0)
APTT	S	3	10	62 (26–200)	64 (32–115)	0 (−6–98)
Fibrinogen	g/L	3	10	1 (0.8–2)	1.1 (0.8–2)	0 (0–0)

Values are median and range

*P*-values from comparisons between IO and CV values and between values at baseline and after haemodilution are presented in Table 5.

**Table 5:** *P*-values (Wilcoxon signed-rank test) from comparisons between simultaneous intraosseous (IO) and central venous (CV) samples at baseline (1) and after haemodilution (2) and between baseline and second samples for both modalities

Variable	IO1 vs CV1	IO2 vs CV2	IO1 vs IO2	CV1 vs CV2
R	0. 008*	0. 01*	0. 8	1
K	0. 5	0. 8	0.07	0.1
α	0.1	0.8	0.03*	0.05*
MA	0.01*	0.4	0.03*	0.01*
LY 30	1	0.6	0.1	0.3
PT	NA	NA	NA	0.01*
APTT	NA	NA	NA	0.5
Fibrinogen	NA	NA	NA	0.01*

Abbreviations: Prothrombin Time, International Normalized Ratio (PT, INR), Activated Partial Thromboplastin Time (APTT), reaction time (R), kinetics (K), α-angle (α), Maximal Amplitude (MA), Lysis at 30 min (LY30)

The MA and α-angle decreased significantly in both IO and CV samples after haemodilution while for the remaining TEG parameters, no significant change was observed. For the standard parameters, only CV samples were compared and an increased PT and a decreased fibrinogen concentration were seen after haemodilution (see Additional files 1 and 2).

## Discussion

If an intraosseous catheter is the only vascular access in a critically ill or injured patient, it is important to know if aspirate from such an access can be used for acute laboratory analyses.

The most frequently studied laboratory parameters are blood gas and acid base status. Kissoon et al. and Abdelmoneim et al. demonstrated similar pH and PCO2 in IO and central venous samples during the earlier phase of cardiopulmonary resuscitation in a porcine model. [20, 21]. We demonstrated previously that analysis of haemoglobin, blood gas and acid base status was feasible using point of care equipment and this was recently repeated in a human study by Veldhoen et al. [22, 23].

Orlowski et al. found similar levels of haemoglobin, electrolytes and blood chemistries in IO, arterial and venous samples [8]. More recently, Miller et al. demonstrated correlation between IO and venous samples for, among others, haemoglobin, glucose, blood urea nitrogen and creatinine [9].

Laboratory analysis of coagulation could be of importance in emergencies where IO access might be used, and in this experiment we studied the possibility of analysing TEG, PT, APTT and fibrinogen in intraosseous and venous samples.

In the majority of the IO samples, clotting in the tubes hindered the analysis of PT, APTT and fibrinogen, while all venous samples were analysed without problems. In the small number of samples available for comparison, clinically relevant differences were observed for APTT but not for PT and fibrinogen.

In the TEG analysis, the reaction time, R, was invariably shorter in the intraosseous than in the venous samples at both baseline and second sampling and the baseline maximal amplitude, MA, was less in the IO samples. The faster activation is in line with the clinically observed hypercoagulability compared with the venous samples. In this context, the lower MA might be partially explained by consumption of coagulation elements in the tube before starting the analysis. However, a lower platelet content in IO samples have been reported previously [13]. Also, the anticoagulant effect of heparin was more readily seen in the venous than in the IO samples when comparing analyses were performed on the same samples with and without heparinase. The reason for this hypercoagulability may be found in the composition of the intraosseous aspirate, but the less straightforward aspiration of the sample could also be a contributing factor [24].

After haemodilution, compared with baseline, a lower TEG maximal amplitude and fibrinogen concentration was seen. There were also an increased PT-INR and a reduced α angle, but the amplitude of these changes were not clinically relevant. These relatively minor changes are consistent with previous findings and reflects the complexity of coagulopathy in trauma and haemorrhage, where haemodilution is considered only to be a part of the problem [18].

This study has several limitations. Firstly, it is an experimental study and results therefore cannot be directly translated to clinical conditions. Secondly, the study is small, and should be considered exploratory in nature, although the main findings appear quite clear. The pig has been demonstrated to be hypercoagulable compared to humans, but since venous samples could be analysed without clotting in these animals, we do not believe that this alone accounts for the findings [17].

Also, haemorrhage and resuscitation, although leading to significant haemodilution, only moderately affected the studied coagulation parameters. It would be of interest to compare IO and venous samples in a setting of more severe coagulopathy although the iatrogenic coagulopathy caused by heparinisation in this study was less apparent in the IO than in the venous samples.

Furthermore, in a clinical scenario where IO access is used, the primary objective is likely to be fluid or drug management. A relevant question is therefore whether infusion in the IO catheter affects agreement between IO and reference laboratory samples. In a previous study, we found IO sample values of haemoglobin, blood gas and acid base parameters to be less reliable when taken from an access with an ongoing infusion. We therefore decided to use IO catheters without infusion in this study and would recommend that any samples for analysis, if possible, are taken before starting an infusion [25].

## Conclusions

Although our sample is small, our observations in this model indicate that the IO samples are hypercoagulable compared to venous samples, which may limit their usefulness for coagulation studies. However, it cannot be excluded that analysis of coagulation in human intraosseous aspirate could perform differently and such data would be useful to further elucidate the subject. It would also be of interest to investigate to what extent sampling technique contributes to the observed hypercoagulability. For example, local heparinisation of the catheters before sampling and reversal with heparinase during TEG analysis might be a possibility. Thus, further studies are necessary to confirm or rule out the possibility of coagulation studies on intraosseous samples.

## Additional files

Additional file 1: Values of Prothrombin Time (PT), Activated Partial Thromboplastin Time (APTT) and Fibrinogen (FIB) in intraosseous (IO) and venous (IV) samples and differences (D) at first (1) and second (2) sampling. (XLSX 9 kb)
Additional file 2: TEG parameters reaction time (R), kinetics (K), α-angle (α), Maximal amplitude (MA) and lysis at 30 minutes (Ly30) in intraosseous (IO) and venous (IV) samples at first (1) and second (2) sampling, and differences. (XLSX 12 kb)

## Abbreviations

APTT: Activated partial thromboplastin time
CTAD: Citrate- theophylline- adenosine- dipyridamole
CV: Central venous
INR: International Normalized Ratio
IO: Intraosseous
IQR: Interquartile range
IU: International Unit
K: Kinetics (TEG)
Ly30: Lysis at 30 minutes (TEG)
MA: Maximal amplitude
MAP: Mean arterial pressure
PT: Prothrombin time
R: Reaction time (TEG)
TEG: Thromboelastography
α: Alpha angle (TEG)
